# The effectiveness of the Liverpool care pathway in improving end of life care for dying cancer patients in hospital. A cluster randomised trial

**DOI:** 10.1186/1472-6963-11-13

**Published:** 2011-01-24

**Authors:** Massimo Costantini, Simona Ottonelli, Laura Canavacci, Fabio Pellegrini, Monica Beccaro

**Affiliations:** 1 Regional Palliative Care Network, National Cancer Research Institute, Genoa, Italy; 2 Regional Bioethics Commission, Tuscany Region, Florence, Italy; 3 Mario Negri Sud Institute, Mario Negri Sud Consortium, Chieti, Italy; 4 Unit of Biostatistics, Casa Sollievo della Sofferenza Scientific Institute, Foggia, Italy

## Abstract

**Background:**

Most cancer patients still die in hospital, mainly in medical wards. Many studies in different countries have shown the poor quality of end-of-life care delivery in hospitals. The Program "Liverpool Care Pathway for the dying patient" (LCP), developed in the UK to transfer the hospice model of care into hospitals and other care settings, is a complex intervention to improve the quality of end-of-life care. The results from qualitative and quantitative studies suggest that the LCP Program can improve significantly the quality of end-of-life care delivery in hospitals, but no randomised trial has been conducted till now.

**Methods and design:**

This is a randomized cluster trial, stratified by regions and matched for assessment period. Pairs of eligible medical wards from different hospitals will be randomized to receive the LCP-I Program or no intervention until the end of the trial. The LCP-I Program will be implemented by a Palliative Care Unit.

The assessment of the end-points will be performed for all cancer deaths occurred in the six months after the end of the LCP-I implementation in the experimental wards and, in the same period of time, in the matched control wards. The primary end-point is the overall quality of end-of-life care provided on the ward to dying cancer patients and their families, assessed using the Global Scale of the Italian version of the Toolkit *"After-death Bereaved Family Member Interview*".

**Discussion:**

This study can be interpreted as a Phase III trial according to the Medical Research Council Framework. In this study, the effectiveness of a fully defined intervention is assessed by comparing the distribution of the endpoints in the experimental and in the control arm.

**Research ID:**

RFPS-2006-6-341619

**Trial registration:**

ClinicalTrials.gov Identifier: NCT01081899

## Background

Despite the development of palliative care services worldwide [1] and numerous studies showing that most cancer patients would prefer to die at home [2,3], most cancer patients still die in hospital [3,4]. Official Italian statistics on place of death are not available for Italy, but according to ISDOC survey [3], it is estimated that one third (34.6%) of cancer patients die in hospital, with broad geographical differences (from 60.2% in the North East to 4.6% in the South and islands). According to the ISDOC survey it is also possible to estimate that about 50% of all hospital cancer deaths occur on medical wards [3].

A number of studies carried out in different countries [5-8] have shown the poor quality of end-of-life care delivery in hospitals. Inappropriate end-of-life care may result in the continuation of invasive treatments that, in addition to having negative consequences in terms of resource management, negatively impact the quality of life of patients [9,10]. The results of two Italian studies conducted in patients who died in hospital from cancer (ISDOC) and other conditions (EOLO) confirm what observed for other countries [11,12].

Since the second half of the 90 s, research groups in the UK and the USA, have developed, implemented and started to evaluate the effect of the introduction of "Care Pathway" for end-of-life care in hospitals [13-15]. The aim of these programs was to introduce the skills necessary to address the complex needs of dying patients and their families into the hospital setting, in an attempt to transfer the "hospice expertise" into a non-specialist context.

A "Care Pathway" is a set of evidence-based, medical and care practices aimed at specific groups of patients. The Care Pathway defines the expected course of events in patient care on an established timeline. Care pathways documentation becomes an integral part of the clinical documentation and allows outcomes assessment [16]. In the healthcare setting, Care Pathways have proved to be useful in improving efficiency and effectiveness of the process of care for various pathologies [17].

Among the different Care Pathways proposed at international level, the most structured and proficient seems to be the "Liverpool Care Pathway for the dying patient" (LCP) [13]. Developed in the UK to transfer the hospice model of care into hospitals and other care settings, it is currently in use in over 20 countries [18]. The implementation of the LCP Program, after an intensive training phase, revolves around the introduction of the LCP medical chart and other specific documentation for patients who have been assessed by a multidisciplinary team to be at the end of their life. The LCP documentation provides recommendations on different aspects of care including comfort measures, anticipatory prescription of medication and the assessment of nonessential medical treatments. It also provides guidance for the psychological and spiritual support of patients and their families. The LCP documentation allows the monitoring of results and supports the implementation of audit procedures.

The Italian version of the LCP - Un percorso integrato per le cure di fine vita in ospedale (LCP-I) - has been developed by the Regional Palliative Care Network of the National Cancer Research Institute of Genoa (Italy) in compliance with the original format. The final version has been endorsed by the Central Team UK, Marie Curie Palliative Care Institute Liverpool (MCPCIL).

In Italy, the LCP-I Program has been successfully piloted in 2007 in the Medical Ward of "Villa Scassi Hospital" in Genoa. The implementation process has been evaluated using a combined qualitative and quantitative approach. Focus groups, performed on sample of doctors and nurses before and after the implementation of the LCP-I, showed a perception of effectiveness of the Program, particularly in pain management and in communication with patients and their families.

The availability of an effective quality improvement program for the care of dying patients in hospitals is particularly relevant to the healthcare scenario. The LCP-I Program has provided enough evidence to justify a randomized trial to evaluate its effectiveness. Although the core objective of the LCP-I is improving the quality of end of life care for dying patients, the Program targets the healthcare professionals working on the hospital ward. The only feasible method of assessing the effectiveness of this Program is by performing a cluster trial, where hospital wards are randomized to receive (or not to receive) the implementation of the LCP-I Program.

## Methods/Design

### Study design

**Figure 1:**
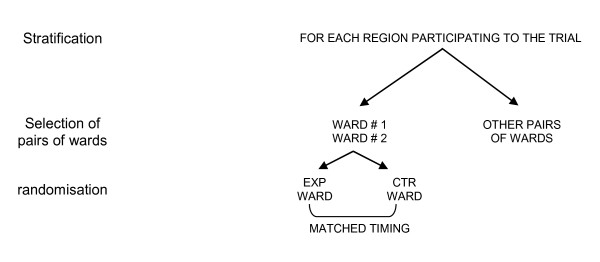
**Selection and matching of pairs of wards participating to the trial**.

This is a randomized cluster trial, stratified by regions and matched for assessment period. Pairs of eligible medical wards from different hospitals will be randomized to receive the experimental intervention (the LCP-I Program) or no intervention until the end of the trial (Figure 1). In each experimental ward, the LCP-I Program will be implemented by a Palliative Care Unit (PCU) responsible for the implementation of the Program on the ward.

### Primary aim

To evaluate the effectiveness of the LCP-I Program in improving the quality of end-of-life care provided to cancer patients who die on hospital medical wards as compared to standard healthcare practices.

### Secondary aims

To evaluate the effectiveness of the LCP-I Program in terms of:

• quality of communication between the healthcare professionals, patients and families;

• quality of emotional support to family members before and after the patients' death;

• coordination of care;

• provision of care focusing on patient's individual needs;

• patient's physical well-being through a better control of physical symptoms;

• appropriateness of therapeutic and diagnostic procedures performed during the last days of the patient's life;

• quality of communication between hospital staff and General Practitioners (GPs).

### General procedures of the cluster trial

**Figure 2:**
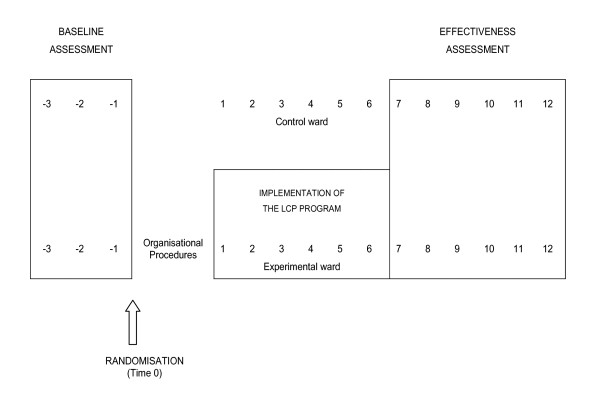
**Design of the assesment procedures**.

Pairs of eligible medical wards from different hospitals will be randomized to receive the experimental intervention (the LCP-I Program) or no intervention at all for the duration of the study (Figure 2). To avoid the risk of contamination, each hospital will be allowed to select only one eligible ward.

Immediately after randomization the PCU responsible for LCP-I implementation on the experimental ward will complete, as quickly as possible, all the organizational procedures required for the implementation of the Program. At the same time the Regional Coordination Structure will start baseline assessment in the experimental and control wards.

Baseline assessment will be performed by evaluating the quality of end-of-life care in each pair of randomized wards for all eligible cancer deaths in the 3 months before the randomization date (months -1 to -3 in Figure 2).

The LCP-I Program will be implemented in the experimental ward by the PCU (t1 to t6, Figure 2). The LCP-I Program has a duration of 6 months from the beginning of the intensive training (months 1-6 in the figure). No intervention will be implemented in the control ward until the end of the evaluation.

Effectiveness assessment (t7 to t12, Figure 2) will be performed by evaluating the quality of end-of-life care in each pair of randomized wards for all eligible cancer deaths occurring in the six months after the conclusion of the LCP-I Program in the experimental ward (months 7 to 12 in Figure 2).

### End-points

Primary and secondary end-points will be evaluated using the same procedure for all eligible cancer deaths, in the baseline and in the effectiveness assessment.

The primary end-point of this trial is the overall quality of end-of-life care provided on the ward to dying cancer patients and their families. The quality of end-of-life care will be assessed using the Global Scale of the Italian version of the Toolkit *"After-death Bereaved Family Member Interview*" [19,20]. The Toolkit is a semi-structured interview with the nonprofessional caregiver closest to the dying patient during the last days of life in hospital. The interview is focused on the patient's last week of life (or less for patients with shorter hospital stays) and on care provided to the caregivers before and after the patients' death.

The secondary aims will be assessed through an analysis of:

1. other six scales from the Toolkit *"After-death Bereaved Family Member Interview"*.

a. Informing and promoting shared decision-making

b. Encouraging advance care planning

c. Focus on individual

d. Attending to the emotional and spiritual needs of the family

e. Providing coordination of care

f. Supporting the self-efficacy of the family

2. Symptom scales from the Italian version of VOICES [21]

a. Pain

b. Breathlessness

c. Nausea and vomiting

3. Diagnostic and therapeutic procedures effectively performed during the last 3 days of life

4. The outcome of an interview with the GPs of patients who died on the wards and who are eligible for the assessment

Furthermore, the evaluation of two leaflets included in the LCP-I Program and delivered to family members after the patient's death is foreseen:

a. "Some useful information", a leaflet delivered to the family members immediately after patient's death.

b. "Facing the loss" a leaflet delivered to bereaved family members during a meeting after the patient's death.

The evaluation of the two leaflets is limited to the families of patients hospitalized in the experimental wards.

### Eligibility criteria

#### Ward level

The inclusion criteria for each medical ward are:

• classified in the regional records as "Medical", "General Medical" or "Internal Medical";

• at least 25 cancer deaths on the ward per year. The data can be estimated from a review of deaths occurring on the ward over a minimum 6 month period during the two years preceding the start of the trial

• consent from the Hospital Management to participate to the trial

• consent from the Head of the Medical Ward to participate to the trial

• consent from an expert and skills-trained PCU to implement the LCP-I Program

Exclusion criteria:

• if in the same hospital another Medical Ward has already been randomly selected to participate in the research program (regardless of which arm was randomized).

#### Individual level

For the two assessment periods (baseline and effectiveness assessment) all cancer patients deceased in the ward during the evaluation period will be considered eligible. ICD- IX criteria will be used to identify cases from the original death certificate. If the patient was a relative of a doctor or a nurse working in the hospital where the ward is located the patient will be considered ineligible.

### Procedures of assessment (Figure 3)

**Figure 3:**
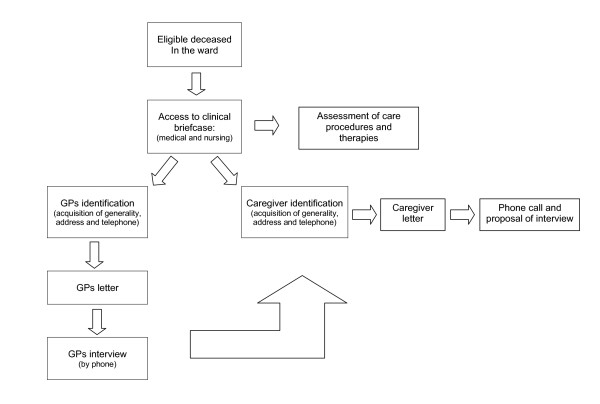
**Procedures of assesment for the eligible population**.

Primary and secondary end-points will be evaluated for all eligible cancer deaths as follows:

**1. Identification of eligible cancer deaths from the ward: **the cancer deaths in the ward will be identified through the hospital's deaths documentation (which lists all deaths regardless of cause). Cancer deaths will be identified by the International Classification of Diseases-Ninth Revision (ICD-IX) criteria, applied to the causes of death as reported in the death certificate of the Italian National Institute of Statistics (ISTAT).

**2. Access to medical and nursing documentation **for the assessment of care procedure

3. Assessment of care procedures

**4. GP identification: **name, address and telephone number of the GPs will be obtained by medical documentation. However, all information are available in the Regional Health records.

**5. Caregiver identification: **information regarding the patients' principal caregiver (name, relationship, address and telephone number), as reported in the medical documentation, must be acquired. The principal caregiver is the person who was closest to the patient during his/her last week of life in hospital. It can be a family member (most frequent) or a friend. If the patient had no caregiver, the interview cannot be performed and the circumstances should be described in detail.

**6. Letter to GPs: **an official letter, signed by the Regional Coordinator, describing the survey and announcing the telephone interview is sent to the deceased' GPs.

**7. GPs telephone Interview: **this must take place within 4 months of the patients death and focuses on two aspects:

◦ communication between ward staff and GPs

◦ confirmation or integration of information regarding the patient's caregiver (name, relationship, address and telephone number).

**8. Final Caregiver identification: **obtained from two main sources: medical documentation and/or patients' GPs.

**9. Caregiver Letter: **an official letter, signed by the Regional Coordinator, introducing the study and announcing the telephone interview is sent to the caregiver.

**10. Phone contact with caregiver: **it occurs 4-5 days after the letter is sent out. The interviewer refers to the contents of the letter and invites the caregiver to participate in an interview at a location of their choice.

**11. Caregiver Interview: **the Caregiver Interview must take place between 2 and 4 months after the patients' death. it should be a face to face interview in a location chosen by the caregiver. Only in exceptional cases where the caregiver is unable to attend the face to face interview (i.e. the caregiver lives in inaccessible location) then the interview may be conducted by phone. When the caregiver is obviously distressed and unable to complete the interview, priority should be given to the section of the Toolkit relative to the overall evaluation (the 6 summary questions).

### Randomisation

Randomization is centralized at the Trial Center of the National Cancer Research Institute (IST) of Genoa. The Regional Coordinators contact the IST Center for the randomization of coupled wards. Randomization lists for each region participating in the trial will be compiled by the Trial Center. The randomization will be carried out for pairs of wards. The sequence of each list will be defined by blocked 1:1 allocation. The randomization of the wards will be conducted by the Regional Coordinators by telephone or fax.

For each request, the trial center:

• verifies the eligibility of the coupled wards

• assigns a numerical code to the two wards

• records the allocation of the wards

## The LCP-I Program

The LCP-I Program is a continuous quality Improvement Program of end-of-life care implemented by a Palliative Care Unit (PCU) in a hospital Medical Ward. The Program is articulated in 10 steps.

### Step 1-3: Development of the implementation project on the ward

• **Step 1: Establishing the project - preparing the environment**

◦ Identify and describe the characteristics of the ward

◦ Identify and describe the characteristics of the PCU

◦ Obtain consent from the subjects involved

◦ Present the general outlines of the LCP-I Program to the ward staff

◦ Outline the LCP-I Program on the ward

◦ Begin the approval procedure for the training program

◦ Register the Project at the National Center for LCP-I

• **Step 2: Developing the Documentation**

◦ Acquire educational materials for training

◦ Prepare the necessary documentation for the ward

• **Step 3: Base review - Retrospective evaluation of variances related to end of life care on the ward**

◦ Review the medical documentation of the patients who died on the ward

◦ Investigate the variances with the ward staff

### Step 4-8: Implementation of the Program on the ward

The implementation of the LCP-I Program, following an intensive training phase, revolves around the introduction of the LCP-I medical chart and other specific documentation for patients who have been assessed by a multi-professional team to be approaching the end of their life. The LCP-I documentation provides recommendations on different aspects of care including comfort measures, anticipatory prescription of medication and the assessment of not appropriate medical and nursing interventions. It also provides guidance for the psychological and spiritual support of patients and their families. The LCP-I documentation allows the monitoring of results and supports the implementation of audit procedures.

• **Step 4: Induction of the Program. Intensive Education Program (duration less than a month)**

◦ 3 modules of 4 hours (12 hours total) repeated twice to allow the participation of all clinical ward staff (doctors and nurses).

• **Step 5: Clinical implementation of the LCP-I documentation. Intensive support to the ward staff (6 weeks)**

◦ the ward staff, closely supported (coaching, telephone and direct guidance, discussion of clinical cases) by the PCU team overseeing the implementation process, starts using the LCP-I documentation for dying patients.

• **Step 6: Semi-intensive support to the ward staff (6 weeks)**

◦ the ward staff, accompanied by the PCU team overseeing the implementation process, learns to use the LCP-I documentation as standard procedure for dying patients. Clinical audits are planned for difficult cases.

• **Step 7: Evaluation and further Training (end of the 4° month)**

◦ the PCU team evaluates the outcome of the preliminary steps with the aim of developing an appropriate training strategy for the ward staff during the subsequent stages of the implementation process.

• **Step 8: Consolidation phase (2 months)**

◦ the LCP-I documentation is established in the ward as an indicator of the quality of end of life care for all dying patients. The PCU team supports the ward staff using the most suitable tools for consolidation of the changes introduced by the LCP-I Program.

### Step 9-10: sustainability of high standards of quality of end of life care on the ward

If the ward staff evaluates positively the use of the LCP-I documentation for dying patients, the PCU responsible for the implementation process, actively supports the development of a sustainable policy for the ward and hospital management. The strategy revolves around the institutionalization of the LCP-I documentation. A feasible approach is the development of a care strategy that guarantees and documents the maintenance of high standards in the quality of end of life care provided.

• **Step 9: Initiation of a strategy for sustainability**

◦ the LCP-I is established as routine procedure on the ward and in the hospital

◦ develop an end-of-life care strategy for the ward.

• **Step 10: Regional and national strategy**

◦ use the outcome of the trial study to stimulate discussions at a regional and national level regarding issues linked to the quality of end-of-life care

### Statistical methods

#### Primary end-point

The primary end-point of this trial is the overall quality of the end-of-life care provided by the ward staff to dying cancer patients and their families. The quality of end-of-life care will be assessed using the Global Scale of the "*Toolkit After-death Bereaved Family Member Interview*".

The Global Scale of the Toolkit is a combination of 6 items that are evaluated at the end of the interview on a 0-10 scale (see page 15 of the Toolkit). The scores of the 6 items are added up and linearized on a 0-100 scale where 0 equals poor quality and 100 equals high quality of care. Where answers are missing for one or more of the 6 items, the score is estimated for the item for which valid answers are available. To calculate the score on the Global Scale a minimum of 4 valid answers out of 6 are necessary.

The quality of the end-of-life care provided to dying patients, assessed with the Global Scale of the Toolkit, is expressed on a 0-100 scale where 0 equals poor quality and 100 equals excellent quality of care.

#### Sample size

An absolute increase of average scores on the Global Scale of the Toolkit of 10-15 points (in experimental wards compared to control wards) can be considered the minimum difference of clinical interest which justifies the implementation of the LCP -I Program. This increase, based on the results of the pilot study corresponds to an Effect Size (ES) of about 0.4. In order to estimate sample size in a cluster trial, along with Type I and II errors, two elements are essential: the intra-cluster correlation coefficient and the average size of the cluster (number of cluster deaths) [22]. So, for this trial:

◦ the intraclass correlation coefficient can be estimated between 0.01 and 0.05 [23]

◦ according to the review of cancer deaths on the Medical Wards potentially involved, we can expect an average number of cancer deaths in the 6 months of the assessment ranging between 15 and 20.

◦ it is foreseen that the regions participating will be able to randomize 10 pairs of Medical Wards (20 clusters overall).

Data in the Table 1 intersect three ICC scenarios (from 0.01 to 0.1) with 4 scenarios of average cluster size (from 10 to 25). In the corresponding sections the total cluster required to achieve a cluster trial with a coefficient of 80% are reported to a two-tailed alpha error of 0.5 and an a hypothetical ES of 0.4. Table 1 reports different sample size scenarios necessary to detect an ES of 0.4 with alpha = 0.05 and a power of 80% conditional to the hypothesized levels of ICC (from 0.01 to 0.10) and average size of the clusters (from 10 to 25). According to the information reported in the previous paragraphs, it seems realistic to assume that the study is feasible with a total number of clusters no greater than 20 [22].

**Table 1:** Total number of clusters required according to different cluster sizes and ICC (ES = 0.4, alpha = 0.05, power = 0.80).

	Average size of the clusters			
	
Intraclass Correlation Coefficient	10	15	20	25
0.01	22	**16**	**12**	**10**
0.05	30	24	**20**	**18**
0.10	38	32	30	28

### Statistical analysis

The centers and patients characteristics will be reported as mean and standard deviation (SD) or frequency and percentage, respectively, for continuous and categorical variables. The distribution of variables of clusters allocated as experimental or control wards will be compared with the t-Student test or one way ANOVA (for ordinal or continuous variables) and with the Pearson Chi-square (for binary or nominal variables).

The assessment of the primary end-point will take into account the clustered design of the trial through the use of hierarchical linear models [24,25]. These models will be adjusted for the average level of quality of care provided to the baseline assessment. The results will be expressed in terms of beta coefficients of regression or in estimated marginal means, 95% Confidence Intervals (IC 95% CI) and level of significance. P-value < 0.05 will be considered statistically significant. The same approach will be used for the assessment of the continuous secondary end-points where there are continual variables. For the assessment of categorical secondary end-points with categorical variables, the hierarchical logistical model will be used.

All the analysis will be conducted using the SAS Statistical Package Release 9.1 (SAS Institute, Cary, NC, USA).

### Ethical issues

Clinical guidelines for the care of dying patients are available but research promoting the acquisition of valid knowledge in this area is still lacking. This can be attributed, in part, to the ethical issues which inevitably arise when carrying out research in this field. The vulnerability of the individuals involved, often aggravated by physical and psychological conditions related to elevated levels of stress, poses serious ethical dilemmas. In addition, dying patients are often unaware of the diagnosis or the gravity of their illness or may be incapacitated. These circumstances, plus difficulties encountered in obtaining valid patient informed consent, constitute barriers which discourage research in the end-of-life care context.

Only a fraction of the above mentioned barriers affects this study. The LCP-I Program is not aimed at increasing knowledge about end-of-life care but at improving the quality of end-of-life care delivered in hospitals. The LCP-I Program introduces in a medical ward effective procedures currently practiced in hospices worldwide onto the hospital ward. Hence, this trial is not aimed at consolidating scientific knowledge regarding the clinical and non-clinical recommendations contained in the Program but rather at assessing the effectiveness of the entire Program as a tool for improving care delivery. In other words, the aim of this study is to assess if the Program succeeds in its intent of improving the quality of end-of-life care delivery on the hospital ward by shifting the attitudes and actions of clinical staff towards better care practices. The study population is the clinical ward staff and not the patients: the involvement of patients and their families is only necessary to verify the efficacy of the change toward a better quality of care delivery.

It is now widely recognized that healthcare institutions, and the medical workforce, have a moral obligation to promote and support interventions for improving medical practices. Although quality improvement activities share some of the characteristics of clinical research and the boundary between these two areas often overlap (in both cases the effectiveness of an intervention is measured) there are, nonetheless, fundamental differences between the two areas that justify the adoption of different procedures for guaranteeing ethical conduct. With consideration to these differences (the minimal risk for the patients involved in quality improvement programs, the demonstrable and immediate benefit to those directly concerned, the introduction of existing evidence-based practices, the flexible and not rigidly formalized protocols, the necessity and obligation for health care institutions to improve the of the quality of care practices, the indirect use of personal data, the absence of sponsors, the clear public interest, etc.), the related international literature highlights the inadequacy of applying the strict protection procedures of clinical research, particularly informed consent, to quality-improvement programs, and favors the adoption of different informative/consent procedures and, where possible, a responsible and informed participation of patients [26].

For the above mentioned reasons, it is evident that it is not feasible to request informed consent from the patients as, in this study, the target population is the clinical staff. However, in order to guarantee confidentiality to patients whose data could be used as indicators to verify the validity of the Program, certain procedures are recommended:

• the ethics committee is required not only to express an opinion on the study, but also to carry out monitoring activities. More precisely, every 3 months the Scientific Coordinator will send to the Ethical Committee of the National Cancer Research Institute of Genoa and to the Ethical Committees of each trust involved in the trial a detailed progress report highlighting all variances, in particular with regards to the relations with the family. For any further clarification, each Ethical Committee can request the Scientific Coordinator's participation.

As far as ethical issues regarding the proper handling of data is concerned it is necessary to distinguish two different phases of the trial:

1. collection and elaboration of data in the patients' medical documentation

2. contact and interview with the caregivers

Regarding collection and elaboration of data from patients' medical documentation:

• the processing of data included in medical documentation is considered of significant public interest, to that purpose, in agreement with Articles 20 and 21 of the Italian Confidentiality Laws, it is accessible to the National Health Service and other Public Health organizations for the planning, management, monitoring and evaluation of healthcare;

• in theory the evaluation could be subject to informed consent of the terminal cancer patients (patients who might die of cancer). In fact, the patients involved in the Program evaluation, who are nearly always incapable of giving valid consent, are identified after death (as cancer deaths), regardless of whether they were hospitalized in a ward where it LCP-I Program was implemented;

• in all cases, where the patients are competent and aware of their health status (parameters previously assessed independently in the LCP-I Program) and where there is considered no risk to the patient, the clinical ward staff will be trained to deliver information to the patient and to ask them the consent to the use of personal data. This procedure will be recorded in the medical records;

• during the study and in subsequent publications, any personal data will be disaggregated and made anonymous.

Regarding contact with the caregivers, the initial contact will be made through a personal letter which includes detailed information and requests their permission to be contacted by telephone. The caregiver will be contacted by phone after at least 3 days and will be invited to participate in the interview. The interview will be conducted by specifically trained staff.

This procedure has been used successfully in the ISDOC survey [21], with very satisfactory results:

• sampling of 2,000 cancer deaths in 30 Italian Local Healthcare Authorities' areas.

• caregiver identification in 95% of cases (N = 1900)

• realization of 1271 valid interviews (64% of the sample).

• reasons for non-completion of the interviews: no caregiver identified (5%), refusal (20%), caregiver not contactable (8%), other (3%).

As described in a published article [21], the interviewers did not encounter particular resistance in the realization of the interviews. Many caregivers found the letter respectful of their circumstances and the information provided about the procedures and the objectives of the study relatively comprehensive. In only one case (out of 1900), a cancer patient's daughter complained that a letter, specifying the cause of her father's death, had been sent to her mother who had not been aware of the nature of her husband illness. Following this observation, the cause of death is not specified in the letter used in this study.

In conclusion, the cluster design of the study and the randomization of wards to receive or not LCP-I Program is necessary for a scientifically based assessment of the effectiveness of the Program. However, to ensure that all the wards involved can benefit from the improvement brought by the Program, unless it proves to be not effective, at the conclusion of the trial the implementation of the LCP-I Program will be proposed also to the control wards.

### Approvals

The study was approved by the Ethics Committee of the National Cancer Research Institute of Genoa (Italy) on September 14^th^, 2009 (Reference number: CCP09.001).

## Discussion

Although it is now widely shared that clinical practice should be, wherever possible, evidence-based, programs to improve the quality of care are often implemented without an evaluation of their effectiveness [27].

Assessing these interventions is a challenge for their complexity, due to the presence of different types of components, activities, interventions and outcomes to be achieved [27], and for specificity of the context of development and application.

The "Medical Research Council's Framework for the Development and Evaluation of Complex Interventions to Improve Health (MRC Framework) [28] is a promising and innovative approach aimed at providing a robust methodological basis to the evaluation of complex interventions. The MRC Framework has been used to develop and evaluate different treatments, services or public health interventions, including programs to improve the quality of care [29-31].

As for as the evaluation of drugs, the MRC Framework is divided into five stages, from "pre-clinical" Phase to Phase IV. Each phase suggests the uses of appropriate quantitative and/or qualitative methodologies for the specific objectives of the phases, and requires a specific study design taking into account the theoretical basis, any evidence on the issue and the context's specificity [29]. More recently, it has been underlined that the MRC Framework is flexible and adaptable approach, and the five phases has not to be clearly separated [32]. For example, "pre-clinical phase" and phases I and II may be part of a unique approach that includes the study and understanding of the problem and context, the construction of models of intervention and the feasibility of assessment [32].

The LCP Program can be defined as a complex intervention to improve the quality of end-of-life care independently of diagnosis and place of death. The results from qualitative studies [33-35] and from experimental assessments in non-randomized trials [36,37] seem to suggest that the LCP Program can improve significantly, and over long-term, the quality of end-of-life care delivery in hospitals and other care settings. According to the MRC Framework, published research on the LCP completed the first three phases, from preclinical phases to phase II.

This study can be interpreted as a Phase III trial according to the MRC framework. In this study, the effectiveness of a fully defined intervention is assessed by comparing the distribution of the endpoints in the experimental arm and in the control arm. Although not all dying patients will be supported by the LCP Program, the assessment is planned on the whole sample of patients deceased in the experimental ward and in the correspondent control ward.

Cluster trial are often used for assessing interventions directed at a hospital team [22]. The validity of this protocol is supported by the results of a recent Cochrane Review [38] on the effectiveness of end-of-life pathways. The review did not find any randomised controlled trials, quasi-randomised trial or high quality controlled before and after studies, to include in the analysis. The authors concluded that there is a lack of evidence supporting such practice [38], and recommend the use of cluster randomised trials as the most effective study design in evaluating this approach [38].

## Competing interests

The authors declare that they have no competing interests.

## Authors' contributions

MC is the principal investigator and the guarantor of this work. We include as co-authors all the members of the Steering group, distinguishing those who wrote significant parts of the paper (in the head of the manuscript) by the others (at the end of the paper as member of the study group). More specifically, MC, SO and MB designed the study protocol and the first draft of this paper; LC and MC wrote the Ethical considerations. MC and FP wrote the statistical aspects of the article. This paper was revised, discussed and emended by all the authors that approved the final version of the manuscript.

## Pre-publication history

The pre-publication history for this paper can be accessed here:

http://www.biomedcentral.com/1472-6963/11/13/prepub
